# Arthroscopically Confirmed Complete Healing of Anterior Cruciate Ligament Avulsion Fracture Following Four‐Corner Pullout Repair Using Ultrahigh‐Molecular‐Weight Polyethylene Tapes: A Case Report

**DOI:** 10.1155/cro/5671446

**Published:** 2026-01-10

**Authors:** Toshiki Kohara, Yuki Okazaki, Shinichi Miyazawa, Takayuki Furumatsu, Yusuke Yokoyama, Masanori Tamura, Koki Kawada, Tsubasa Hasegawa, Tomonori Tetsunaga, Kazuki Yamada, Toshifumi Ozaki

**Affiliations:** ^1^ Department of Orthopedic Surgery, Okayama University Graduate School of Medicine, Dentistry, and Pharmaceutical Sciences, Okayama, Japan, okayama-u.ac.jp; ^2^ Department of Orthopedic Surgery, Tsuyama Chuo Hospital, Tsuyama, Japan, tch.or.jp; ^3^ Center for Education in Medicine and Health Sciences, Medicine, Dentistry and Pharmaceutical Sciences, Okayama University, Okayama, Japan, okayama-u.ac.jp; ^4^ Department of Orthopedic Surgery, NHO Fukuyama Medical Center, Fukuyama, Japan; ^5^ Department of Orthopedic Surgery, Japanese Red Cross Okayama Hospital, Okayama, Japan; ^6^ Department of Musculoskeletal Health Promotion, Faculty of Medicine, Dentistry and Pharmaceutical Sciences, Okayama University, Okayama, Japan, okayama-u.ac.jp; ^7^ Department of Medical Materials for Musculoskeletal Reconstruction, Faculty of Medicine, Dentistry and Pharmaceutical Sciences, Okayama University, Okayama, Japan, okayama-u.ac.jp

## Abstract

We report a case of tibial insertion avulsion fracture of the anterior cruciate ligament (ACL). A 10‐year‐old boy who fell from a skateboard was diagnosed with a tibial insertion avulsion fracture of the ACL and was treated arthroscopically. The avulsed fragment was provisionally fixed with guide pins inserted into its four corners, and the lateral view was checked to avoid penetration of the growth plate. Two ultrahigh‐molecular‐weight polyethylene tapes were passed through the ACL just above its tibial insertion, pulled through the four‐corner bone tunnels in an X‐shaped configuration, and tightened. The patient was immobilized for 3 weeks, and partial weight bearing was initiated at 4 weeks. Bone union was confirmed at 6 months using plain radiographs, and second‐look arthroscopy and implant removal were performed 8 months postoperatively. During arthroscopy, complete union with smooth continuity of the articular cartilage at the fracture site and a stable ACL were observed. The patient had a full knee range of motion and no pain at the final follow‐up.

## 1. Introduction

Tibial insertion avulsion fractures of the anterior cruciate ligament (ACL) are relatively common injuries in children, occurring in approximately 3 out of 100,000 children annually [1]. According to the Meyer–McKeever classification (Type I, nondisplaced; Type II, partially displaced with a hinged posterior cortex; Type III, completely displaced) [2], nondisplaced or minimally displaced fractures can be treated conservatively. However, displaced or unstable fractures require surgical treatment to prevent nonunion and residual instability [2]. Various arthroscopic surgical techniques have been reported, including Kirschner wire fixation [3], screw fixation [4–7], pullout suture fixation [8–11], and suture anchor fixation [12], all of which have shown favorable clinical outcomes. In children and adolescents with open growth plates, fixing the fracture without penetrating the physis is preferable [12]. Various materials, including strong nonabsorbable sutures [8–11] and tapes [13], have been described for pullout suture fixation. However, no reports exist on second‐look arthroscopic evaluation following fixation of ACL tibial insertion avulsion fractures in patients with open growth plates.

Herein, we report the case of a 10‐year‐old boy diagnosed with an ACL tibial insertion avulsion fracture. The patient was treated arthroscopically with pullout suture fixation to avoid damage to the growth plate and achieve complete healing of the articular cartilage with a good clinical outcome.

## 2. Case Presentation

A 10‐year‐old boy complained of left knee pain after falling from a skateboard. He presented to our hospital 9 days after the injury. Physical examination revealed knee effusion and tenderness with a restricted range of motion (ROM). The Lachman and anterior drawer tests were positive. Initial plain radiographs showed a tibial insertion avulsion fracture of the ACL (Figure 1a,b), with no evidence of comminution on computed tomography (Figure 1c). The fragment was completely displaced, and the injury was categorized as Type III. Magnetic resonance imaging confirmed an avulsion fracture of the ACL at the tibial insertion with no additional bone marrow edema beyond the fracture site (Figure 1d). The patient was immobilized with a long‐leg splint for pain control until surgery.

Figure 1Preoperative imaging findings. (a) Anteroposterior and (b) lateral radiographs showing a tibial insertion avulsion fracture of the anterior cruciate ligament (ACL). (c) Three‐dimensional computed tomography reconstruction showing a tibial insertion avulsion fracture of the ACL (white dotted circle) as a single piece without comminution. (d) Magnetic resonance imaging (T2‐weighted image) showing a tibial insertion avulsion fracture of the ACL (white dotted circle).

**Figure:**
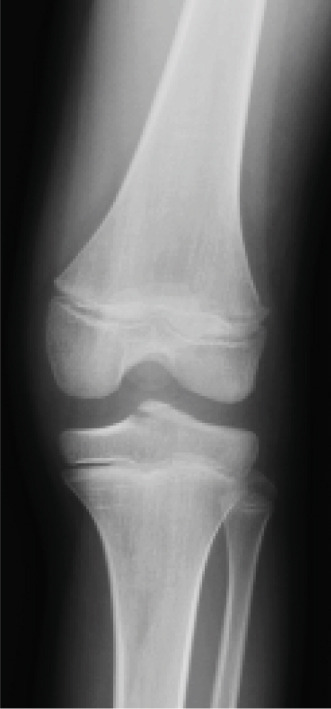
(a)

**Figure:**
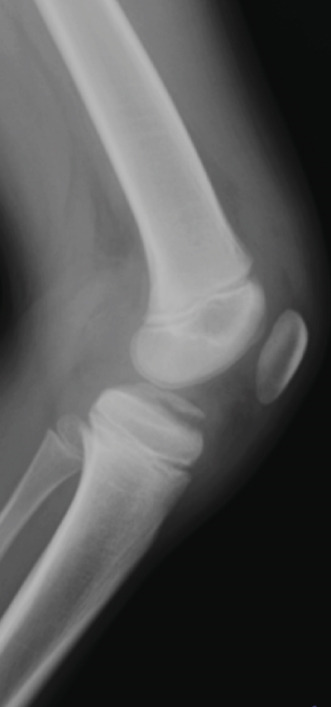
(b)

**Figure:**
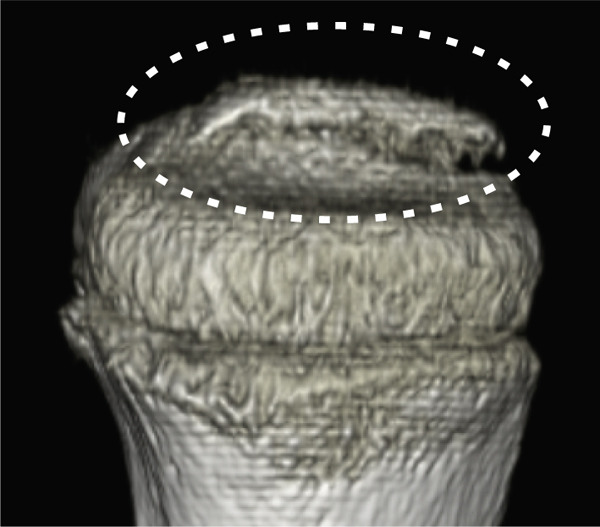
(c)

**Figure:**
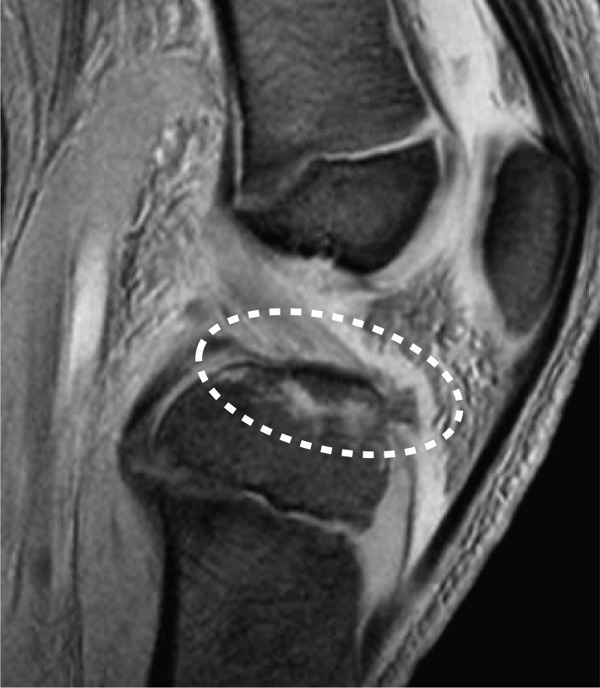
(d)

Arthroscopic reduction and fixation were performed under general anesthesia 17 days after the injury. An avulsed fragment was identified at the tibial insertion of the ACL (Figure 2a). No tears in the midsubstance of the ACL, meniscal tears, or moderate‐to‐severe cartilage damage were observed. Fracture debris, hematoma, and synovium around the fracture site were debrided using a shaver. The bone fragment was reduced to the bone bed using the tip of the ACL tibial drill guide. A 2.5‐cm longitudinal incision was made medial to the tibial tubercle, and the subcutaneous tissues were retracted to expose the bone. Four 2.4‐mm guide pins were inserted into the four corners around the bone fragment (anteromedial/lateral and posteromedial/lateral corners) through the ACL tibial drill guide, and the fragment was provisionally fixed (Figure 2b). This procedure was performed under fluoroscopic guidance, and the lateral view was examined to avoid growth plate penetration. Two ultrahigh‐molecular‐weight polyethylene (UHMWPE) tapes with a width of 2.0 mm (Ultratape; Smith & Nephew, London, UK) were passed through the anterior and posterior portions of the ACL just above the tibial insertion using a Knee Scorpion suture passer (Arthrex, FL, USA) (Figure 2c). The anterior suture was pulled through the posterior drill holes using a suture retriever (Smith & Nephew), whereas the posterior suture was crossed anteriorly to the ACL and pulled through the anterior drill holes (Figure 2d). Fracture reduction and stability were confirmed in the extended and flexed positions using an image intensifier and arthroscopy during suture tensioning. Finally, the sutures were manually tightened during knee extension using a 12‐mm suture button (Arthrex) (Figure 3). Although the ideal position is directly on bone, the button was placed over the patellar tendon. To create the two lateral bone tunnels from the anteromedial surface of the tibia, the guide pins must be inserted at a very shallow angle, which is technically challenging. Therefore, the button was positioned over the patellar tendon.

Figure 2Intraoperative arthroscopic findings. (a) The tibial avulsion fragment of the ACL was identified. (b) 2.4‐mm guide pins were inserted into the four corners (anteromedial/lateral and posteromedial/lateral corners) of the fragment using an ACL tibial drill guide. (c) Two ultrahigh‐molecular‐weight polyethylene tapes (Ultratape; Smith & Nephew) were passed through the anterior and posterior portions of the ACL using a Knee Scorpion suture passer. (d) The tape inserted into the anterior portion of the ACL was pulled into a drill hole created in the posteromedial/lateral corners of the ACL using a suture retriever, and the tape inserted into the posterior portion of the ACL was pulled into a drill hole created in the anteromedial/lateral corners of the ACL. ACL: anterior cruciate ligament.

**Figure:**
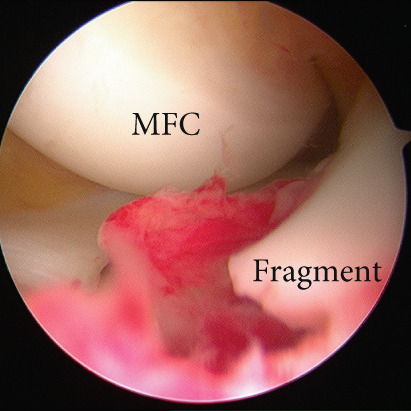
(a)

**Figure:**
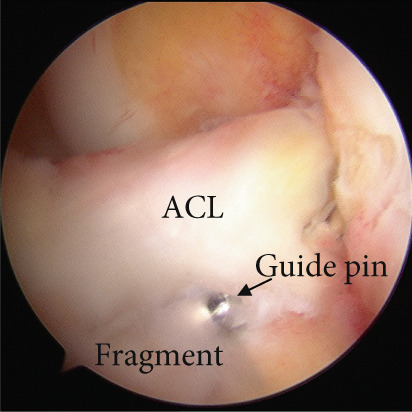
(b)

**Figure:**
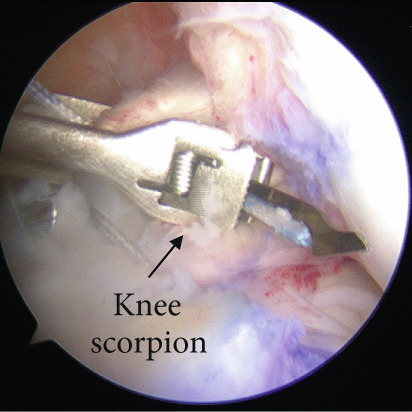
(c)

**Figure:**
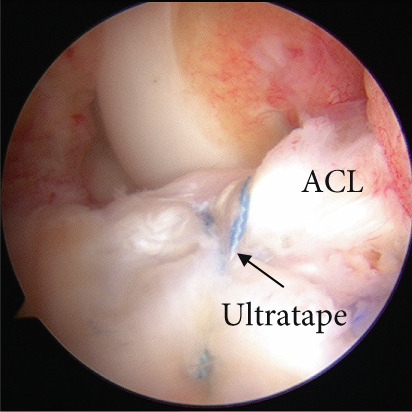
(d)

Figure 3Postoperative radiographs at primary surgery and a schematic diagram. The bone tunnels created in the anteromedial/lateral corners (blue lines) and posteromedial/lateral corners (red lines) of the anterior cruciate ligament are shown. (a) Anteroposterior radiograph. (b) Lateral radiograph. (c) Schematic diagram. Blue line: anterior suture, red line: posterior suture.

**Figure:**
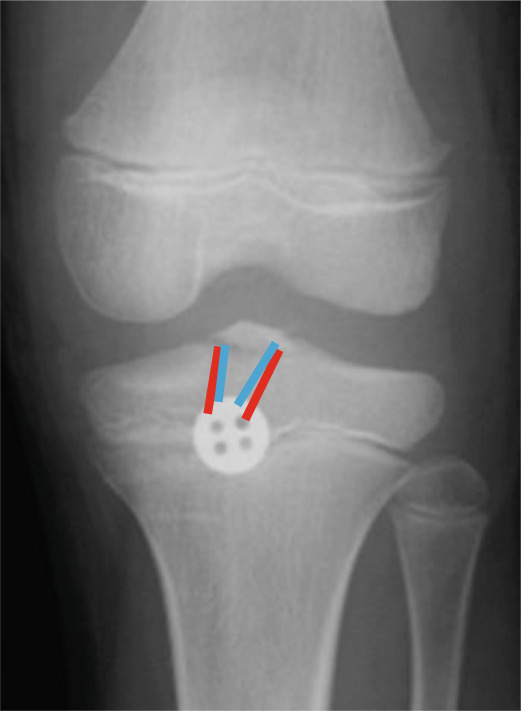
(a)

**Figure:**
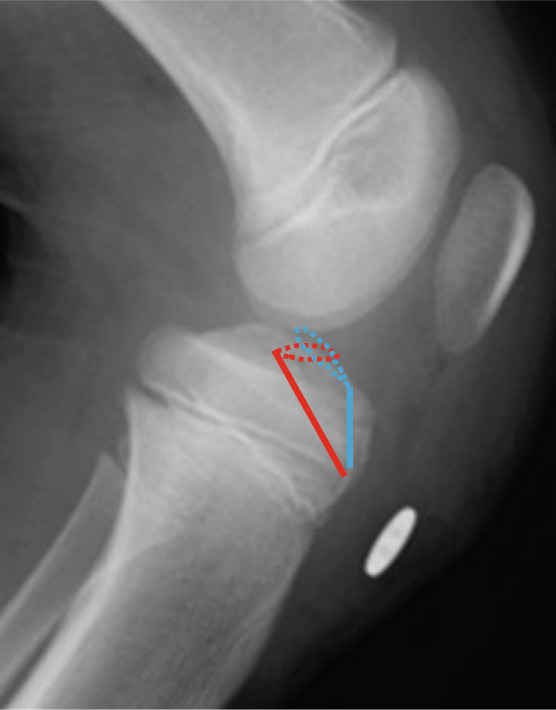
(b)

**Figure:**
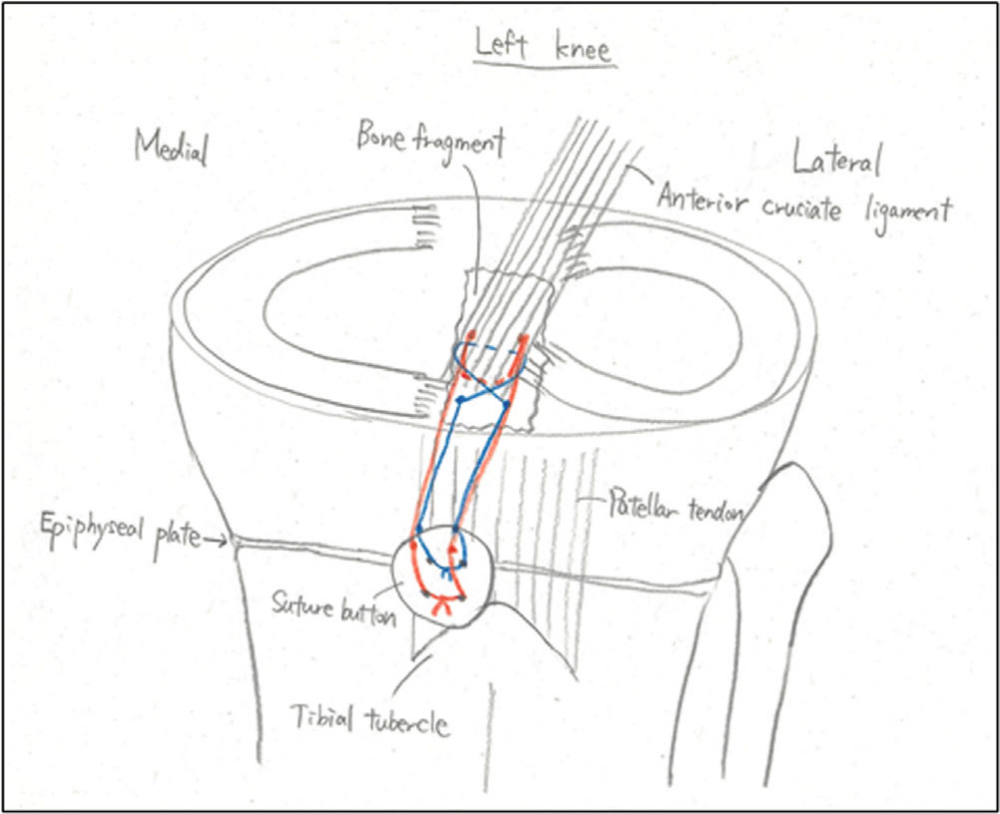
(c)

Postoperatively, the knee was immobilized using a knee brace for 3 weeks. ROM exercises, limited to 90° of flexion, began 3 weeks after surgery. Toe‐touch weight bearing was permitted on the day after surgery, and partial weight bearing was initiated at 4 weeks. Full weight bearing and unrestricted ROM exercises were permitted 6 weeks postoperatively. Bone union was confirmed using plain radiography at 6 months, and second‐look arthroscopy and implant removal were performed 8 months after the primary surgery. During arthroscopic evaluation, the fracture was completely united and covered with cartilage, and the ACL appeared stable (Figure 4a). Abnormal synovial hyperplasia was observed in the anterior femoral intercondylar region, which was resected using a shaver (Figure 4b). At the last follow‐up, 3 years after the primary surgery, the patient had no symptoms in daily activities and regained full knee ROM. Both Lachman and pivot‐shift tests yielded negative results. The patient′s pain visual analog scale score was 0, the Tegner score was 6, the Lysholm score was 100 points, and the International Knee Documentation Committee score was 100 points (Figure 5).

Figure 4Second‐look arthroscopic findings 8 months after the primary surgery. (a) Abnormal synovial hyperplasia was observed in the intercondylar notch of the femur. (b) The complete union of the tibial avulsion fracture of the ACL and the stability of the ACL were observed. ACL: anterior cruciate ligament.

**Figure:**
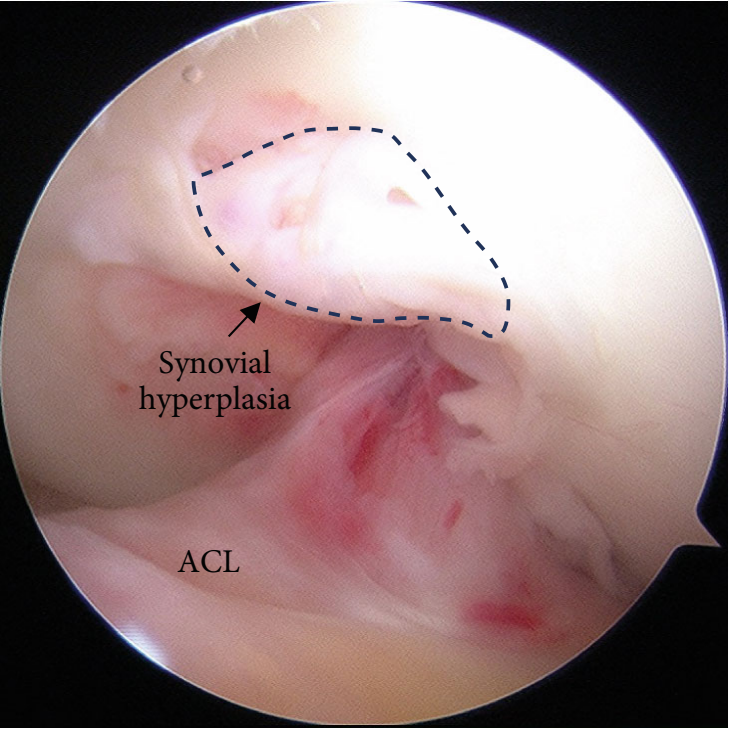
(a)

**Figure:**
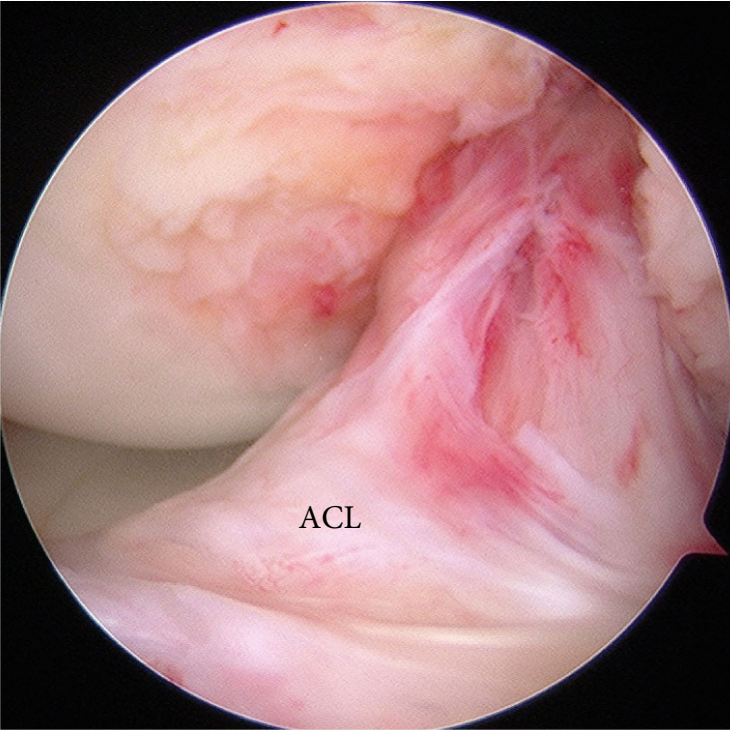
(b)

Figure 5Postoperative radiographs 3 years after the primary surgery. (a) Anteroposterior and (b) lateral views showed no evidence of osteoarthritis.

**Figure:**
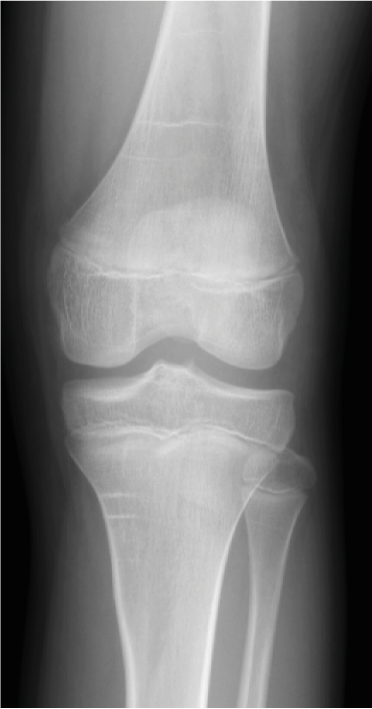
(a)

**Figure:**
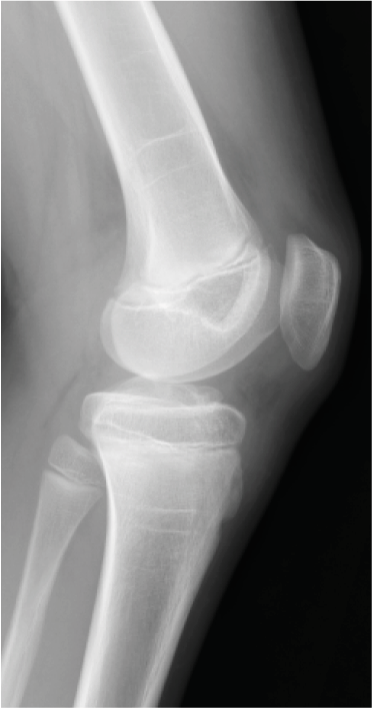
(b)

## 3. Discussion

According to the Meyers and McKeever classification, Type I and Type II fractures can be treated conservatively [14]. However, Type III fractures require surgical treatment to prevent ACL insufficiency and loss of knee motion [15]. Traditionally, open reduction and internal fixation have been performed [16]; however, less invasive arthroscopic surgery has recently become more common. Various arthroscopic fixation techniques have been reported, including screw fixation [4–7], suture anchor fixation [8–11], and pullout suture fixation [12]. In a cadaveric study, Bong et al. reported that pullout suture fixation using three No. 2 FiberWire sutures (Arthrex) had a higher load‐to‐failure than antegrade screw fixation using a 4‐mm cannulated cancellous screw with a washer (319 vs. 129 N) [17]. UHMWPE tape has been shown to have a higher maximum load‐to‐failure than conventional threads [18]. Based on these findings, UHMWPE tapes were used for pullout fixation in this case.

There are two unique techniques for pullout suture fixation: one that preserves the growth plate and another that penetrates it. Mylle et al. reported a case of antegrade screw fixation that penetrated the growth plate, resulting in anterior epiphysiodesis and tibial hyperextension [19]. A meta‐analysis revealed cases of angular deformity and leg length discrepancy > 1 cm in skeletally immature patients, occurring at rates of 1.4% and 2.8%, respectively, after ACL reconstruction [20]. Therefore, a technique that preserves the growth plate is preferable.

Moreover, sutures need to be tightened not on the bone but on the patellar tendon and retinaculum, which may result in relatively lower fixation strength; therefore, a robust pullout suture fixation technique is necessary. In this case, two sutures were passed medially and laterally to enhance the fixation stability against multidirectional forces while increasing the compressive force on the bone fragment. In addition, sutures were passed through the four corners of the bone fragment to compress the fragment as a surface and improve reduction quality. In cases involving small fragments or comminuted fractures, pulling through the fragment can be technically challenging, which limits the indications for this technique.

In this case, the clinical and radiological outcomes were satisfactory. This report is the first to include second‐look arthroscopy following pullout suture fixation of a tibial insertion fracture of the ACL; arthroscopic evaluation confirmed cartilage coverage at the fracture site and excellent ACL quality. In addition, this evaluation helps address postoperative cartilage or meniscal injuries. However, this study has some limitations. First, the tensile force applied during the tightening was not measured. Although the stability of the fragment was confirmed in both flexed and extended positions, the exact tensile force remains unclear. Second, the follow‐up period was relatively short. Koch et al. reported a case of physeal‐sparing ACL reconstruction that resulted in varus deformity and a 20‐mm overgrowth. Deformities and leg‐length discrepancies are possible until the growth plates close; therefore, long‐term follow‐up is needed [21].

## 4. Conclusion

A tibial insertion avulsion fracture of the ACL was treated using the four‐corner pullout technique with UHMWPE tapes, successfully preserving the growth plate, and complete healing was confirmed during second‐look arthroscopy. This technique provides strong fixation while avoiding complications related to the growth plates.

## Ethics Statement

This case report was conducted in accordance with the Declaration of Helsinki. Institutional review board approval was not required for a single case report. Written informed consent for publication was obtained from the patient and his parents.

## Conflicts of Interest

The authors declare no conflicts of interest.

## Funding

This research did not receive any specific grant from funding agencies in the public, commercial, or not‐for‐profit sectors.

## Data Availability

Data are available from the corresponding author upon reasonable request.
